# Role of SIRT1 in Hepatic Encephalopathy: In Vivo and In Vitro Studies Focusing on the NLRP3 Inflammasome

**DOI:** 10.1155/2021/5522708

**Published:** 2021-10-12

**Authors:** Fangzhou Jiao, Yao Wang, Qian Chen, Pan Cao, Chunxia Shi, Maohua Pei, Luwen Wang, Zuojiong Gong

**Affiliations:** Department of Infectious Disease, Renmin Hospital of Wuhan University, Wuhan, China

## Abstract

Hepatic encephalopathy (HE) is a neuropsychiatric disorder resulting from acute or chronic liver failure. This study is aimed at investigating the therapeutic effects and mechanisms of SIRT1 in thioacetamide- (TAA-) induced rat HE models. A selective activator (CAY10602) and inhibitor (EX527) of SIRT1 were used in this study. All male rats were separated into control, TAA, CAY10602+TAA, and EX527+TAA groups. Histological damage, liver function, serum ammonia, behavioral changes, and brain oxidative stress were measured in each group. Western blotting was used to measure SIRT1, NLRP3, ASC, and IL-1*β* protein expression. The results showed that CAY10602 alleviated liver injury, improved neurological decline, reduced microglial activation and brain oxidative stress, and improved the survival rates of HE rats. Moreover, CAY10602 inhibited activation of the NLRP3 inflammasome in microglia of the brain cortex in HE rats. Next, cell experiments confirmed that CAY10602 inhibited activation of the NLRP3 inflammasome in BV2 microglial cells. However, inhibition of SIRT1 by EX527 or lentivirus could enhance activation of the NLRP3 inflammasome in this process. Finally, CAY10602 reduced the neurotoxicity induced by high levels of ammonia in HT22 cells. Taken together, CAY10602 alleviates TAA-induced HE by suppressing microglial activation and the NLRP3 inflammasome and reducing the neurotoxicity of NH_4_Cl in HT22 cells. A pharmacologic activator of SIRT1 may be a promising approach for the treatment of HE.

## 1. Introduction

Hepatic encephalopathy (HE) is a dangerous neuropsychiatric disorder resulting from acute or chronic liver failure. A previous study found that liver failure patients with HE grades III and IV had the worst 30-day survival rate (2.4%) compared to patients with HE grades I and II (26.1%) and those without HE (39.7%) [1]. The clinical manifestations of HE include disturbance of consciousness, abnormal behavior, and coma. At present, several factors are involved in the pathogenesis of HE, while neuroinflammation and hyperammonemia are considered key factors [2]. Recent studies have indicated the importance of inflammasome activation in various neuroinflammatory and neurodegenerative diseases. Several cells of the CNS express inflammasome components, including microglia, neurons, and astrocytes. Persistent activation of microglia has been proposed to lead to neuronal cell death and promote the process of neuroinflammation. The current understanding of NLRP3 inflammasome activation in microglia indicates that it plays an important role in neuroinflammation [3]. In addition to neuroinflammation, increased ammonia is a key factor in the pathogenesis of hepatic encephalopathy. The direct toxicity of excess ammonia on neurons has been reported [4]. One study found that ammonia inhibited cell viability and increased ROS levels in SH-SY5Y cells and cerebellar granule neurons [5]. These studies suggested that NLRP3 inflammasome activation and hyperammonemia might be involved in the pathogenesis of HE.

Sirtuins are class III histone deacetylases and include seven members (SIRT1–7). Among these sirtuins, SIRT1 is well studied and considered a key regulator involved in a number of biological processes, including circadian rhythms, inflammation [6], reprogramming, aging, tumorigenesis [7], and metabolism [8]. Many studies have demonstrated that SIRT1 is closely linked with inflammatory reactions. A previous study found that resveratrol inhibits NLRP3 inflammasome activation in sepsis-associated encephalopathy and in microglia [9]. One recent study demonstrated that resveratrol improves memory impairments and motor deficits in moderate-grade hepatic encephalopathy rats. The study only measured SIRT1 activity in the hippocampus, and the precise molecular mechanisms were not assessed in this study [10]. As a natural compound, some studies have reported that resveratrol may activate SIRT1 activity indirectly [11] and has other functions in addition to its effects on SIRT1 [12].

In this study, we investigated the effects of SIRT1 on thioacetamide-induced hepatic encephalopathy in male rats by using the SIRT1 activator CAY10602 and inhibitor EX527. Next, to identify the mechanisms by which SIRT1 alleviates hepatic encephalopathy by regulating neuroinflammation, we further explored the effects of SIRT1 on NLRP3 inflammasome activation in BV2 microglial cells. Finally, we assessed the effects of SIRT1 on the neurotoxicity of NH_4_Cl in HT22 neuronal cells. These findings provide a promising approach for the treatment of hepatic encephalopathy.

## 2. Materials and Methods

### 2.1. Chemicals and Drugs

Thioacetamide (TAA, 163678), LPS (L2880), ATP (FLAAS), N-acetyl-L-cysteine (NAC, A9165), and NH_4_Cl (A9434) were purchased from Sigma–Aldrich (USA). H_2_O_2_ (H112515) was purchased from Aladdin (China). CAY10602 was purchased from MedChemExpress (USA). EX527 was purchased from Selleckchem (USA). Anti-SIRT1 (#8469), anti-NLRP3 (#15101), anti-IL-1*β* (#12426), anti-cleaved caspase-3 (#9661), and anti-GAPDH (#5174) antibodies were obtained from Cell Signaling Technology (USA). Anti-ASC (sc-514414) was purchased from Santa Cruz Biotechnology (USA). An anti-Iba-1 antibody was purchased from Abcam (ab178847, USA).

### 2.2. Animal Treatment

**Figure 1:**
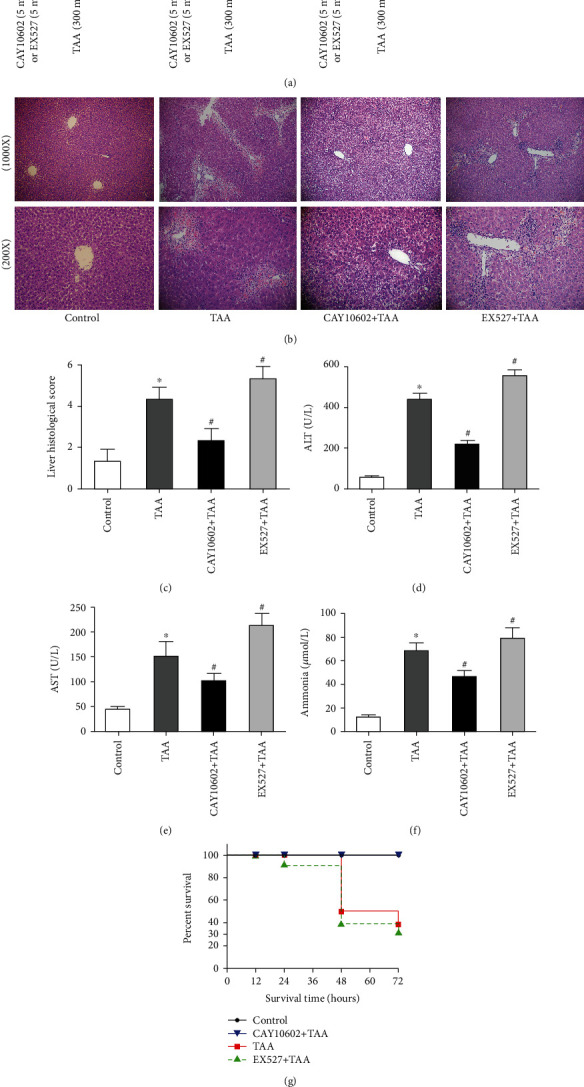
Effect of CAY10602 or EX527 on liver injury in HE rats. (a) Schematic illustrations of experimental procedures. (b) The histological changes of liver. (c) The liver histological score of liver. (d) The serum levels of ALT. (e) The plasma levels of AST. (f) The plasma levels of ammonia. (g) The survival rates of rats in 72 h. ^∗^*P* < 0.05, compared with the control group. ^#^*P* < 0.05, compared with the TAA group. The data represent the means ± SD.

Male Wistar rats (5–6 weeks, 130–180 g) were obtained from Beijing Vital River Laboratory Animal Technology. Animal Care and Use Committee of Renmin Hospital of Wuhan University approved the animal use and care protocols (SYXK (E) 2015–0027), which were designed in compliance with the guidelines of National Institutes of Health about Care and Use of Laboratory Animals. In this study, HE induced by TAA was extensively proven to be a well-characterized animal model of HE [13]. Briefly, the rats were randomly divided into four groups (*n* = 10 each): control, TAA, CAY10602 plus TAA, and EX527 plus TAA groups. HE model rats were administered 300 mg/kg/day TAA by intraperitoneal injection for 3 days [14]. The dosage of EX527 was 5 mg/kg following the description outlined in a previous study [15]. The appropriate dosage of CAY10602 (5 mg/kg) was selected according to previous dosage experiments. The CAY10602 plus TAA and EX527 plus TAA groups were intraperitoneally administered CAY10602 (5 mg/kg) and EX527 (5 mg/kg), respectively, for 3 days, which were administered 2 h before every dose of TAA. The control group was also given the same doses of DMSO solution. After 24 h of the last dose of TAA, all rats were anesthetized with 1.5% isoflurane and sacrificed, and livers, brains, and serum were harvested (Figure 1(a)).

### 2.3. Biochemical Analysis

Blood samples were collected from rats, and the serum levels of ALT and AST were measured by using a Hitachi automatic analyzer (Japan). The serum ammonia concentration in the samples was determined by an Ammonia Assay Kit (Sigma–Aldrich, USA) as previously described [16]. The ammonia reaction with *α*-ketoglutarate and NADPH is catalyzed by L-glutamate dehydrogenase and forms L-glutamate and NADP+. The decrease in absorbance at 340 nm in this reaction was measured.

### 2.4. Histopathological Examination

Histological examination of the samples was performed by a standard protocol. Briefly, the tissues were embedded in paraffin, sectioned at 5 *μ*m, and stained with hematoxylin and eosin. Histopathological changes in the liver were analyzed under a light microscope (Olympus, Japan). The liver histological score was used to evaluate injury in the liver tissues [17]. In this scoring system, no necrosis was counted as 0, mild necrosis (<10% of area) was counted as 1, moderate necrosis (10–25% of area) was counted as 2, and severe necrosis (>25% of area) was counted as 3. No inflammation was counted as 0, <10% of area as 1, 10–50% of area as 2, and >50% of area as 3.

### 2.5. Behavioral Testing

The open field test (OFT) was performed to evaluate the locomotor activity and exploratory behavior of rats [18]. Each rat was gently placed in the center of a dimly illuminated rectangular cage (120 × 90 × 35 cm^3^), and spontaneous activity was observed for 5 min through an automated video tracking system (Ethovision XT 11.5).

The frequencies of rearing, total moving distance, and average velocity were quantified by this system. The cage was thoroughly cleaned with 75% alcohol before the next rat test. The Y-maze test was performed to evaluate the spatial orientation learning ability of rats [19]. The Y-maze test apparatus included three closed plastic arms with an angle of 120° between them. Each rat was allowed to explore the maze for 5 minutes. Arm entry of rat was recorded. Three consecutive entries of the rats into three different arms were termed spontaneous alteration behavior. The movement locus in the arms was recorded and analyzed using Ethovision XT 11.5. The percentage of alterations in behavior was calculated as the number of entries into three arms consecutively/(total number of arms entered–2) × 100. The percentage of alterations in behavior was used to measure spatial orientation learning ability.

### 2.6. Assessment of Brain Oxidative Stress Marker Activities

The lipid peroxidation product level of MDA was estimated by using a thiobarbituric acid-reactive substance (TBARS) assay. The absorbance was measured at 534 nm. The reduced GSH level was measured according to the method of Beutler et al. [20]. The absorbance was measured at a wavelength of 412 nm.

### 2.7. Immunofluorescence Staining of Iba-1

Immunofluorescence staining of the microglial marker Iba-1 was used to explore microglial activation. Briefly, after being blocked with BSA, the sections were incubated with a rabbit polyclonal anti-Iba-1 antibody overnight at 4°C. Then, the sections were incubated at 37°C for 60 min with a goat anti-rabbit secondary antibody. Afterward, DAPI was used to stain the nuclei of the cells for 5 min. The immunofluorescence results were calculated by automatic fluorescence microscope.

### 2.8. Double Immunofluorescent Staining of Brain Tissue

The protein expression of CD11b and NLRP3 in brain tissues was detected by double immunofluorescent staining. Briefly, after being blocked for 1 h, brain slices were incubated overnight with an anti-NLRP3 antibody (1 : 100). After that, the sections were incubated overnight with another primary antibody (anti-CD11b antibody, 1 : 100). Then, the sections were incubated with fluorescent secondary antibody (1 : 100) at room temperature for 0.5 h. DAPI was used to stain the nuclei. The image results were recorded with automatic fluorescence microscope.

### 2.9. Cell Culture and Treatments

BV2 microglial cells, HEK293T cells, and HT22 neuronal cells were obtained from the China Center for Type Culture Collection (CCTCC) and cultured in DMEM medium at 37°C containing 5% CO_2_. LPS and ATP were used to establish the activation of the NLRP3 inflammasome cell model in BV2 microglial cells [21, 22]. Briefly, BV2 cells were treated with LPS (10 *μ*g/ml) alone for 5 h and then treated with ATP for 1 h. In addition, in HT22 cells, high levels of ammonia in neuronal cells were induced by NH_4_Cl according to a previous study [4].

### 2.10. Cell Viability Assay

Cells were seeded into a 96-well plate (1 × 10^4^ cells/well). For BV2 cells, CAY10602 (0.5, 1, 4, 8, 10, 15, 20, and 40 *μ*M) or EX527 (1, 5, 10, 20, 40, 80, 160, and 320 *μ*M) was added to the cells for 6 h. For HT22 cells, different concentrations of CAY10602 or EX527 were added for 24 h. Then, 10 *μ*L of CCK-8 dye (Cell Counting Kit-8 assay, Dojindo, Japan) was added to the cells for 1 h. After that, the optical density at 450 nm was recorded by microplate reader.

### 2.11. Lentiviral Transduction

The SIRT1 shRNA plasmid (pLVshRNA-EGFP(2A)-puro-SIRT1) and negative control (NC) plasmid (pLVshRNA-EGFP(2A)-puro) were obtained from Miaoling Bioscience & Technology (China) and contained puromycin-resistance and EGFP genes. The SIRT1 shRNA plasmid and packaging plasmids (pSPAX2 and pMD2.G) were transfected into HEK293T cells by Lipofectamine 2000. The genes and protein expression of SIRT1 were used to measure SIRT1 knockdown efficiency.

### 2.12. Measurement of Intracellular ROS

After treatment, cells were incubated in 10 *μ*M DCFH-DA-containing medium in the dark at 37°C for 30 min. Then, cells were washed with serum-free culture medium three times to remove DCFH-DA. The cells were digested by trypsinization and resuspended in cold PBS. Then, the level of ROS was measured by DCF fluorescence with flow cytometry. The level of ROS was measured by the mean fluorescence intensity in each group.

### 2.13. Quantitative RT–PCR Assay

Sample RNA was extracted by using the TaKaRa RNA Extraction Kit (Japan) according to the manufacturer's instructions. Then, total RNA (1 *μ*g) was reverse-transcribed to cDNA by the Prime Script™ RT reagent Kit with gDNA Eraser (Takara, Japan). Quantitative RT–PCR was performed using PCR analysis (Applied Biosystems, USA). The PCR protocol was carried out as follows: initial activation at 95°C for 30 s, followed by 40 cycles of denaturation at 95°C for 5 s, 60°C for 30 s, and annealing at 60°C for 34 s. Gene levels were determined with the 2^−*ΔΔ*Ct^ method. All gene primers were as follows: SIRT1, forward, 5′-GGA GCA GAT TAG TAA GCG GCT TG-3′ and SIRT1, reverse, 5′-GTT ACT GCC ACA GGA ACT AGA GG-3′; IL-1*β*, forward, 5′-AGA GCA TCC AGC TTC AAA TC-3′ and IL-1*β*, reverse, 5′-CGG AGC CTG TAG TGC AGT TGT C-3′; and GAPDH, forward, 5′-ATG GGT GTG AAC CAC GAG A-3′ and GAPDH, reverse, 5′-CAG GGA TGA TGT TCT GGG CA-3′.

### 2.14. Flow Cytometric Analysis for Apoptosis

HT22 cells were seeded in 6-well culture plates. After treatment, cells were collected and resuspended in binding buffer (PE Apoptosis Detection Kit, USA). Next, the cells were maintained with PE (5 *μ*l) and 7-AAD (10 *μ*l) in DMEM medium for 15 min in the dark. The apoptosis rate in HT22 cells was measured by flow cytometric analysis.

### 2.15. Western Blotting

After treatments, sample protein was extracted from the tissues or cells. Sample protein concentrations were detected by the BCA assay (China). The extracted proteins were separated on 12% SDS–PAGE gels and then transferred to a PVDF membrane (USA). After that, the membranes were maintained with 5% milk for 1 h and followed by incubation overnight with specific primary antibodies at 4°C. The antibodies were anti-SIRT1, anti-NLRP3, anti-IL-1*β*, anti-ASC, anti-cleaved caspase-3, and anti-GAPDH. After incubation, bands were incubated with secondary antibody for 1 h. The bands were visualized and quantified by the Odyssey Imaging system.

### 2.16. Statistical Analysis

Data are represented as the mean ± standard deviation. The significant differences of two groups were analyzed using Student's *t*-test. The differences among multiple groups were analyzed using one-way ANOVA. The statistical process was conducted by SPSS v17.0. *P* value <0.05 was considered statistically distinct.

## 3. Results

### 3.1. CAY10602 Alleviated Liver Injury and Improved Survival Rates in HE Rats

Liver histological examination showed massive hemorrhagic necrosis, liver lobules, and inflammatory cell infiltration in HE group rats. CAY10602 pretreatment ameliorated TAA-induced tissue damage. However, EX527 enhanced liver damage compared with the HE groups (Figure 1(b)). Moreover, CAY10602 decreased the histological score of the liver, and EX527 increased the scores compared with the HE groups (Figure 1(c)). Similarly, CAY10602 also alleviated liver injury in female rats (Fig. 1S). In addition, we evaluated the levels of hepatic enzymes and ammonia in each group. CAY10602 significantly reduced the levels of ALT, AST, and ammonia, while EX527 enhanced the levels of hepatic enzymes and ammonia (Figures 1(d)–1(f)). Finally, the survival rate of each group of rats was observed. The results showed that 40% of rats survived in the HE group, whereas only 30.0% survived in the EX527 group. All CAY10602 group and control group rats survived (Figure 1(g)).

### 3.2. CAY10602 Alleviated Neurological Decline and Brain Oxidative Stress and Reduced Microglial Activation in the Cortex and in the Hippocampus

**Figure 2:**
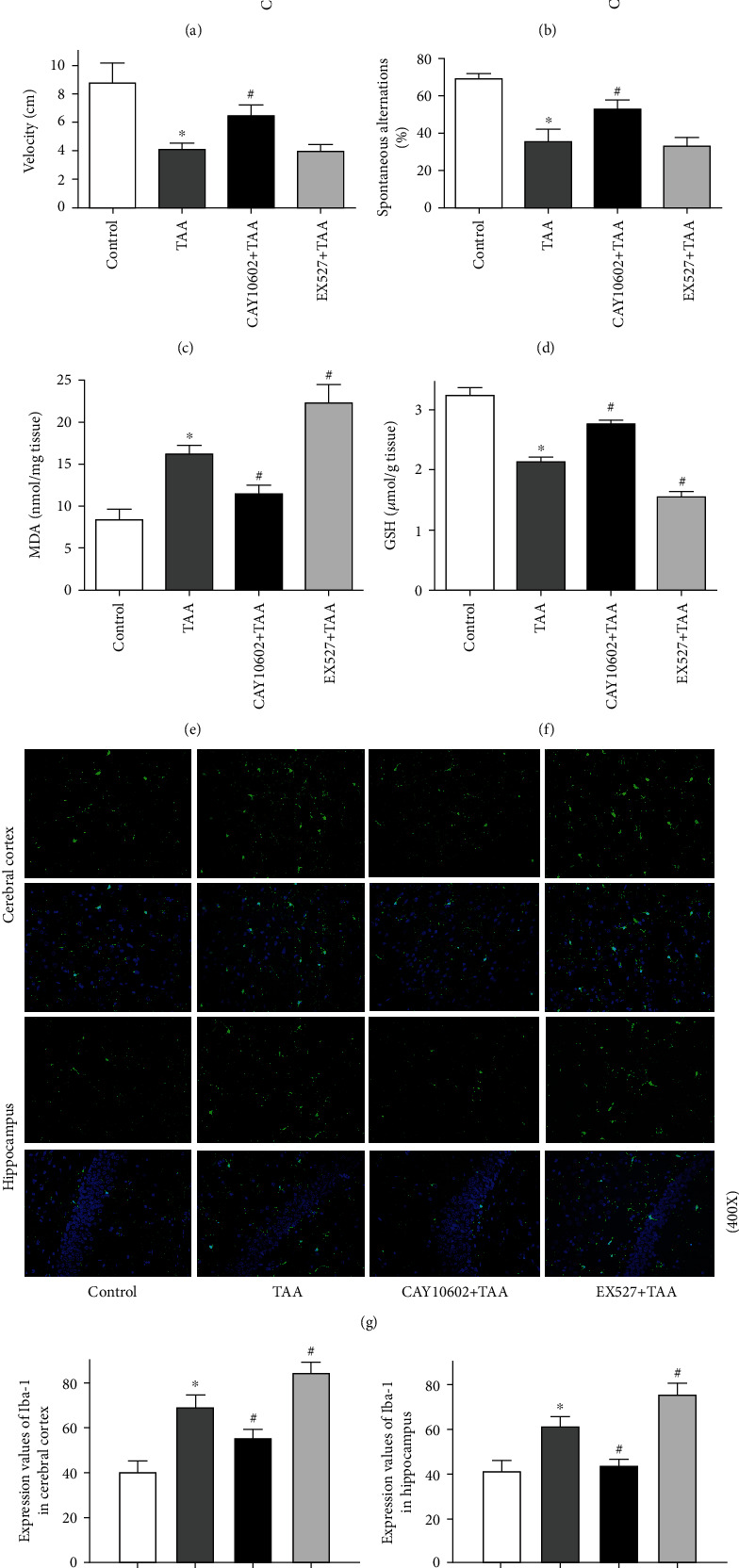
Effect of CAY10602 or EX527 on the behavioral testing, brain oxidative stress marker activities, and microglia activation in HE rats. OFT was performed to evaluate the locomotor activity and exploratory behavior of rats. (a) The frequencies of rearing. (b) Total moving distance. (c) Average velocity. The percentage of alteration behavior of Y-maze test was used to measure spatial orientation learning ability. (d) Alteration behavior. (e) The MDA and (f) GSH level in brain cortex. (g) Immunofluorescence staining of Iba-1, a marker of microglia activation. (h) The expression values of Iba-1 in the cerebral cortex. (i) The expression values of Iba-1 in hippocampus. ^∗^*P* < 0.05, compared with the control group. ^#^*P* < 0.05, compared with the TAA group. *n* = 5 per group. The data represent the means ± SD.

The locomotor activity and exploratory behavior of rats were measured by OFT. As shown in Figure 2, compared to the control, the frequencies of rearing, total moving distance, and average velocity were decreased in the HE groups. CAY10602 administration alleviated the changes in behavior in TAA rats (Figures 2(a)–2(c)). However, the EX527 administration group showed similar results compared with the HE groups. The spatial orientation learning ability of rats was measured by the Y-maze test. CAY10602 improved the changes in behavior in TAA rats (Figure 2(d)). In addition, brain oxidative stress marker activities were determined. CAY10602 administration resulted in a significant decrease in the brain cortex MDA content compared to that in the TAA group, while EX527 increased the contents of MDA. Likewise, CAY10602 resulted in a significant increase in the brain cortex contents of GSH compared to the TAA group, while EX527 reduced the levels of GSH (Figures 2(e) and 2(f)). In addition, we assessed the activation of microglia in brain tissue. As a specific marker of microglia, immunofluorescence staining results showed that Iba-1 expression in the cerebral cortex and hippocampus was markedly elevated in the HE groups and EX527 administration group. However, CAY10602 pretreatment downregulated the activation of microglia compared with that in the HE groups (Figures 2(g)–2(i)).

### 3.3. CAY10602 Inhibited Activation of the NLRP3 Inflammasome in the Brain Cortex

**Figure 3:**
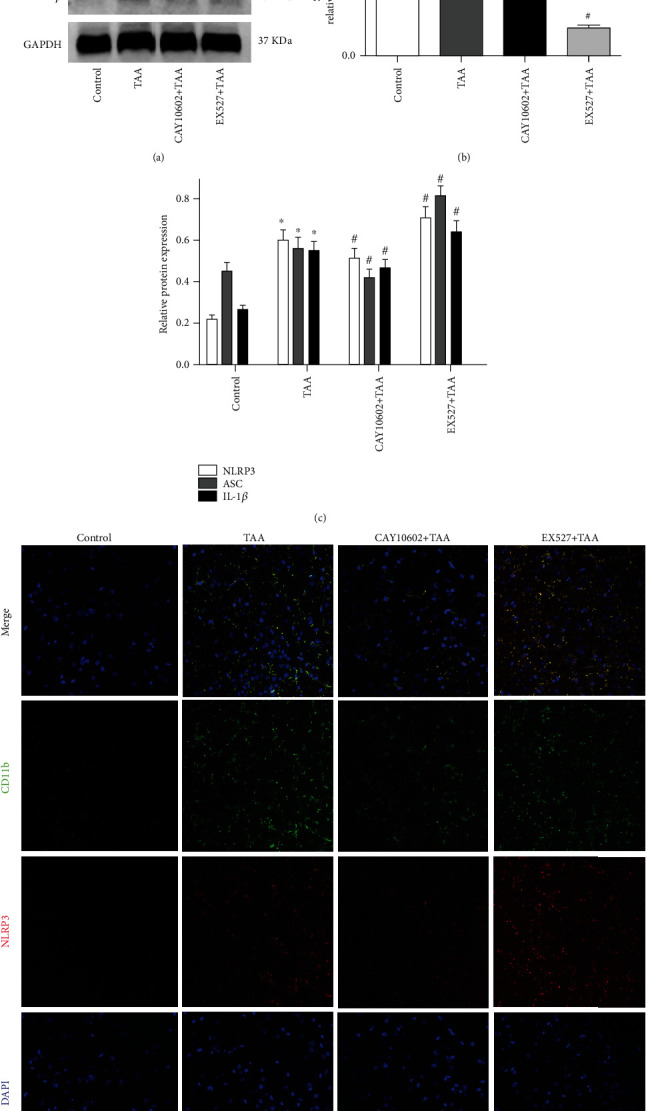
Effect of CAY10602 or EX527 on the activation of NLRP3 inflammasome in the cerebral cortex. (a) The protein expressions of SIRT1, NLRP3, ASC, and IL-1*β* were analyzed by western blotting. (b) The quantitative blots of SIRT1. (b) The quantitative blots of NLRP3, ASC, and IL-1*β*. (d) Immunofluorescence of NLRP3 (red) and CD11b (green) proteins. ^∗^*P* < 0.05, compared with the control group. ^#^*P* < 0.05, compared with the TAA group. *n* = 5 per group. The data represent the means ± SD.

To investigate the effects of CAY10602 in the HE rat model, we evaluated the effect of CAY10602 on the NLRP3 inflammasome in the brain cortex. The protein expression levels of SIRT1 NLRP3, ASC, and IL-1*β* were analyzed by western blotting (Figure 3(a)). The results showed that the expression of NLRP3, ASC, and IL-1*β* was significantly elevated in the HE groups. CAY10602 decreased the expression of these proteins. However, EX527 increased their expression in this process (Figures 3(a) and 3(c)). To identify the cell types which express the NLRP3 in HE brain tissue, immunofluorescence assay was used to detect the expression of NLRP3 (red) and the microglial marker CD11b (green). Similar to the western blot results, CAY10602 reduced the coexpression of NLRP3 and CD11b in brain tissue after TAA stimulation, while EX527 increased their expression (Figure 3(d)). These results showed that CAY10602 suppressed activation of the NLRP3 inflammasome in microglia of the HE brain cortex.

### 3.4. LPS- and ATP-Induced NLRP3 Inflammasome Activation Decreased SIRT1 Levels in BV2 Microglial Cells

**Figure 4:**
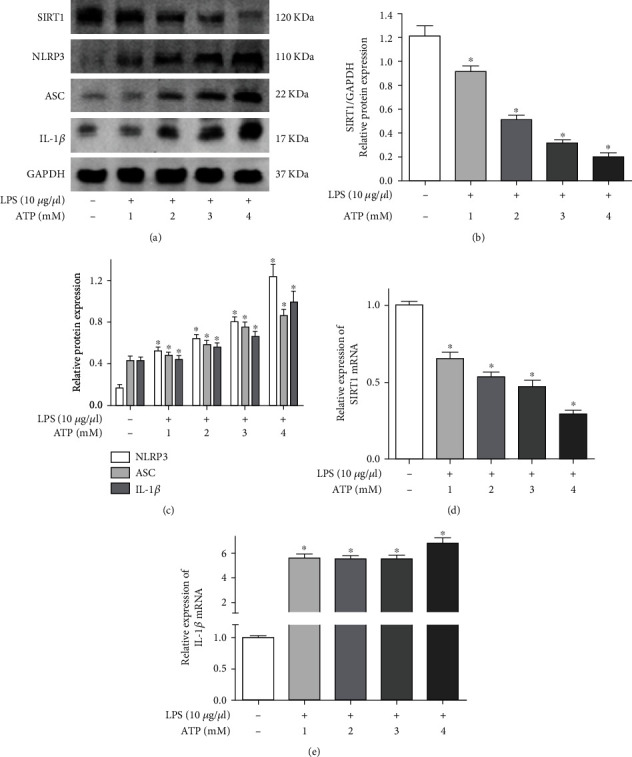
LPS- and ATP-induced NLRP3 inflammasome activation and decreased SIRT1 in BV2 microglial cells. (a) Cells were exposed to normal saline, 10 *μ*g/ml LPS and ATP (1, 2, 3, and 4 mM). The protein expression of SIRT1, NLRP3, ASC, and IL-1*β* was detected by western blotting. (b) The quantitative blots of SIRT1. (c) The quantitative blots of NLRP3, ASC, and IL-1*β*. (d) The gene expression of SIRT1. (e) The gene expression of IL-1*β*. ^∗^*P* < 0.05, compared with the control group. *n* = 5 per group. The data represent the means ± SD.

To determine the suitable drug dosage, we assessed the effect of LPS (10 *μ*g/ml) and ATP (1, 2, 3, and 4 mM) on NLRP3 inflammasome activation in BV2 cells. As shown in Figure 4(a), compared with the control group, LPS and ATP significantly increased the protein expression of NLRP3, ASC, and IL-1*β* in a dose-dependent manner. However, SIRT1 expression was decreased in a dose-dependent manner (Figure 4(b)). Similar results were found at the gene level, and LPS and ATP treatment suppressed the mRNA expression of SIRT1 in a dose-dependent manner compared with the control group (Figure 4(d)). However, the mRNA levels of IL-1*β* in BV2 cells were significantly increased after LPS and ATP treatment (Figure 4(e)). According to these results, the drug dosages of LPS (10 *μ*g/ml) and ATP (4 mM) were used for further experiments.

### 3.5. CAY10602 Alleviated Activation of the NLRP3 Inflammasome in BV2 Microglial Cells

**Figure 5:**
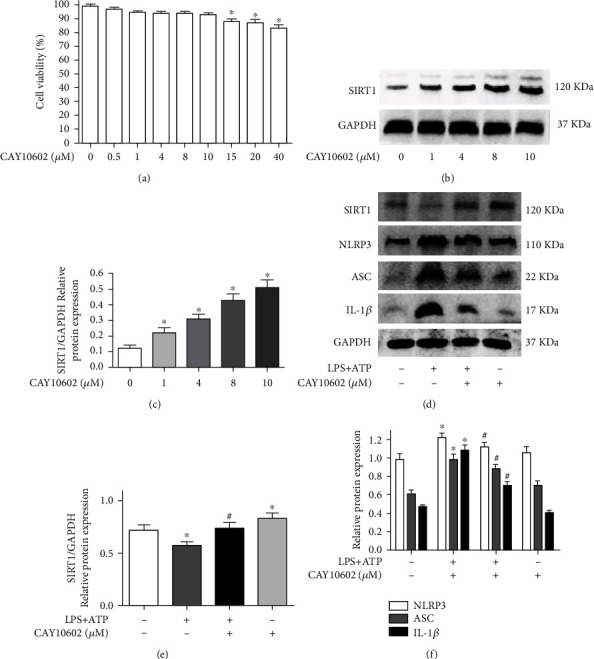
CAY10602 alleviated the activation of NLRP3 inflammasome in BV2 cells. (a) The cell viability of BV2 cells at different concentrations CAY10602. (b) BV2 cells exposed to CAY10602 (1, 4, 8, and 10 *μ*M) for 8 h; then, the protein expression of SIRT1 was measured by western blotting. (c) The quantitative blots of SIRT1. (d) Cells were exposed to normal saline, 10 *μ*g/ml LPS+4 mM ATP, 10 *μ*g/ml LPS+4 mM ATP+10 *μ*M CAY10602, and 10 *μ*M CAY10602. The protein expression of SIRT1, NLRP3, ASC, and IL-1*β* was detected by western blotting. (e) The quantitative blots of SIRT1. (f) The quantitative blots of NLRP3, ASC, and IL-1*β*. ^∗^*P* < 0.05, compared with the control group; ^#^*P* < 0.05, compared with the control+10 *μ*g/ml LPS+4 mM ATP group. *n* = 5 per group. The data represent the means ± SD.

Nontoxic doses of CAY10602 were assessed by CCK-8 assays. We explored the cytotoxicity of CAY10602 on BV2 cells at different concentrations (0.5, 1, 4, 8, 10, 15, 20, and 40 *μ*M). The cell viability was lower than 90% when the dose of CAY10602 was 15 *μ*M (Figure 5(a)). Thus, concentrations of 1, 4, 8, and 10 *μ*M CAY10602 were selected to measure the activation effect on SIRT1. The results showed that CAY10602 upregulated the protein expression of SIRT1 in a dose-dependent manner in BV2 microglial cells (Figures 5(b) and 5(c)). Next, we explored the effect of CAY10602 (10 *μ*M) on LPS- and ATP-induced NLRP3 inflammasome activation. Western blotting results showed that CAY10602 increased the expression of SIRT1 (Figure 5(e)) and decreased the expression of NLRP3, ASC, and IL-1*β* in BV2 cells after LPS and ATP treatment (Figure 5(f)).

### 3.6. EX527 Enhanced Activation of the NLRP3 Inflammasome in BV2 Microglial Cells

**Figure 6:**
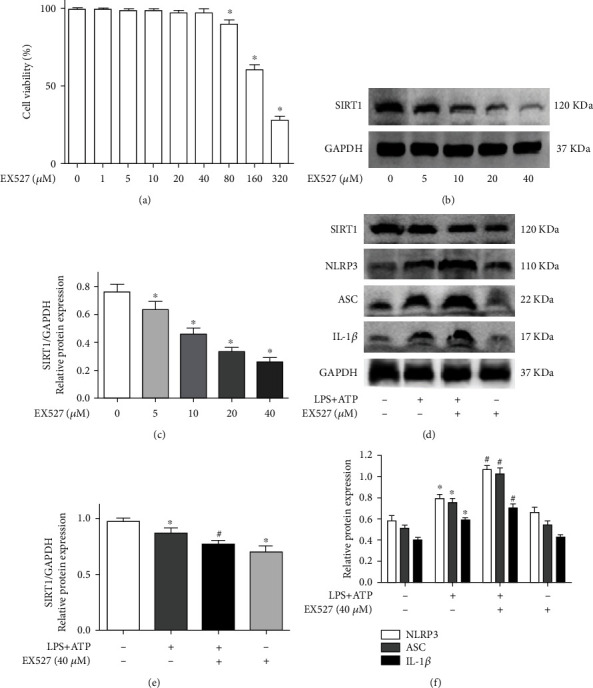
EX527 enhanced the activation of NLRP3 inflammasome in BV2 microglial cells. (a) The cell viability of BV2 cells at different concentrations EX527; (b) BV2 microglial cells exposed to EX527 (5, 10, 20, and 40 *μ*M) for 8 h; then, the protein expression of SIRT1 was measured by western blotting; (c) the quantitative blots of SIRT1; (d) cells were exposed to normal saline, 10 *μ*g/ml LPS+4 mM ATP, 10 *μ*g/ml LPS+4 mM ATP+40 *μ*M EX527, and 40 *μ*M EX527. The protein expression of SIRT1, NLRP3, ASC, and IL-1*β* was detected by western blotting; (e) the quantitative blots of SIRT1; (f) the quantitative blots of NLRP3, ASC, and IL-1*β*. ^∗^*P* < 0.05, compared with the control group; ^#^*P* < 0.05, compared with the control+10 *μ*g/ml LPS+4 mM ATP group. *n* = 5 per group. The data represent the means ± SD.

First, we explored the cytotoxicity of EX527 on BV2 cells by CCK-8 assay. The cell viability was lower than 90% when the dose of EX527 was 80 *μ*M (Figure 6(a)). Thus, EX527 concentrations of 5, 10, 20, and 40 *μ*M were selected to measure the inhibitory effect on SIRT1. As shown in Figures 6(b) and 6(c), EX527 inhibited SIRT1 in a dose-dependent manner in BV2 microglial cells. Next, we explored the effect of EX527 (40 *μ*M) on LPS- and ATP-induced NLRP3 inflammasome activation. We analyzed the expression of SIRT1, NLRP3, ASC, and IL-1*β* by western blotting and found that EX527 further decreased the expression of SIRT1 (Figure 6(e)) and further enhanced the expression of NLRP3, ASC, and IL-1*β* in BV2 cells after LPS and ATP treatment (Figure 6(f)).

### 3.7. SIRT1 Knockdown Enhanced Activation of the NLRP3 Inflammasome

**Figure 7:**
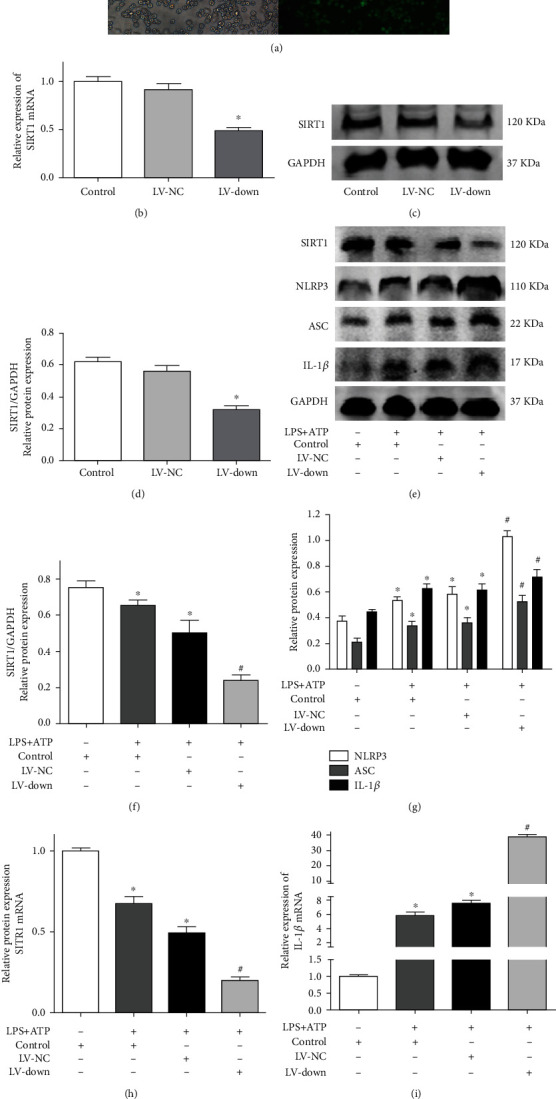
SIRT1 knockdown enhanced the activation of NLRP3 inflammasome. (a) The SIRT1 knockdown lentiviral vector (LV) was transfected into BV2 microglial cells. The expression of EGFP was observed by fluorescence microscope; (b) the mRNA of SIRT1 of SIRT1 knockdown cells was determined by RT–PCR; (c) the protein expression of SIRT1 of SIRT1 knockdown cells was determined by western blotting; (d) the quantitative blots of SIRT1; (e) cells were divided into four groups: control, control+10 *μ*g/ml LPS+4 mM ATP, LV-NC+10 *μ*g/ml LPS+4 mM ATP, and LV-down+10 *μ*g/ml LPS+4 mM ATP group. The protein expression of SIRT1, NLRP3, ASC, and IL-1*β* was detected by western blotting; (f) the quantitative blots of SIRT1; (g) the quantitative blots of NLRP3, ASC, and IL-1*β*; (h) the gene expression of SIRT1; (i) the gene expression of IL-1*β*. ^∗^*P* < 0.05, compared with the control group. ^#^*P* < 0.05, compared with the LV-NC+10 *μ*g/ml LPS+4 mM ATP group. *n* = 5 per group. The data represent the means ± SD.

After the SIRT1 knockdown lentiviral vector (LV) was transfected into BV2 microglial cells, the expression of EGFP was observed by fluorescence microscopy (Figure 7(a)). SIRT1 knockdown efficiency of BV2 microglial cells was determined by RT–PCR and western blotting. The results showed that the LV-down group showed an obviously decreased mRNA and protein expression of SIRT1 compared with the LV-NC group (Figures 7(b)–7(d)). In addition, there was no significant difference in SIRT1 expression between the control and LV-NC groups. Next, we found that the expression of SIRT1 was decreased in the control and LV-NC groups by LPS and ATP stimulation. However, the LV-down group further decreased the expression of SIRT1 compared with the LV-NC group (Figures 7(e) and 7(f)). Finally, we measured the expression of NLRP3 inflammasome proteins (NLRP3, ASC, and IL-1*β*) in each group. The results showed that the expression of these proteins was further increased in the LV-down group compared with the LV-NC group (Figure 7(g)). Moreover, the mRNA levels of SIRT1 and IL-1*β* were detected by RT–PCR. Similarly, compared with the LV-NC group, the LV-down group showed a further decrease in SIRT1 mRNA levels and an increase in IL-1*β* mRNA levels (Figures 7(h) and 7(i)).

### 3.8. SIRT1 Knockdown Increased ROS Levels in LPS- and ATP-Induced BV2 Microglial Cells

**Figure 8:**
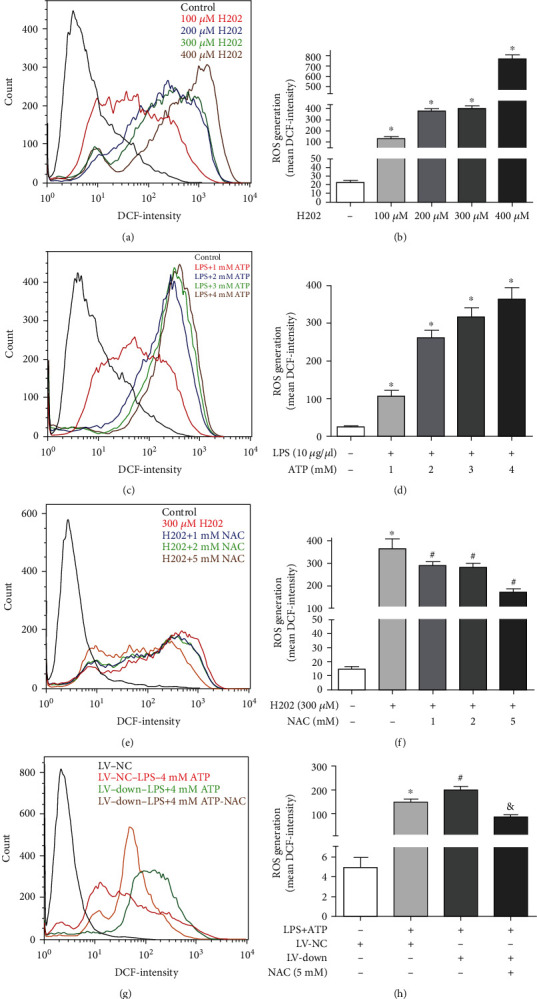
SIRT1 knockdown increased the ROS levels in LPS- and ATP-induced BV2 microglial cells. The ROS levels were measured by DCF fluorescence with flow cytometry. (a) Cells were treated with H_2_O_2_ at different concentrations (100, 200, 300, and 400 *μ*M, respectively); (b) the mean DCF-intensity in each group; (c) cells were exposed to normal saline, 10 *μ*g/ml LPS and ATP (1, 2, 3, and 4 mM); (d) the mean DCF-intensity in each group; (e) cells were treated with normal saline, H_2_O_2_ (300 *μ*M), and H_2_O_2_ (300 *μ*M)+NAC (1, 2, and 5 mM); (f) the mean DCF-intensity in each group; (g) cells were divided into four groups: LV-NC, LV-NC+10 *μ*g/ml LPS+4 mM ATP, LV-down+10 *μ*g/ml LPS+4 mM ATP, and LV-down+10 *μ*g/ml LPS+4 mM ATP+NAC (5 mM); (h) the mean DCF-intensity in each group. In Figure 4(f), ^∗^*P* < 0.05 versus control. ^#^*P* < 0.05, compared with the H_2_O_2_ (300 *μ*M). In Figure 4(h), ^∗^*P* < 0.05 versus LV-NC. ^#^*P* < 0.05 versus LV-NC+10 *μ*g/ml LPS+4 mM ATP. ^&^*P* < 0.05 versus LV-down+10 *μ*g/ml LPS+4 mM ATP. In others, ^∗^*P* < 0.05 versus control. *n* = 5 per group. The data represent the means ± SD.

Intracellular ROS was measured by using DCFH-DA. To select the suitable dosage of H_2_O_2_, we assessed the level of ROS in BV2 cells at different concentrations (100, 200, 300, and 400 *μ*M). As shown in Figures 8(a) and 8(b), H_2_O_2_ administration significantly enhanced ROS levels in a dose-dependent manner. Similarly, we found that ROS levels were obviously increased in LPS- and ATP-induced activation of the NLRP3 inflammasome (Figures 8(c) and 8(d)). In addition, pretreatment with NAC (1, 2, and 5 mM) reduced the high level of ROS induced by H_2_O_2_ (300 *μ*M) stimulation, especially at 5 mM NAC (Figures 8(e) and 8(f)). As shown in Figures 8(g) and 8(h), compared with the LV-NC group, the ROS levels were obviously increased in the LV-down group after LPS and ATP treatment. However, the LV-down plus NAC group showed obviously decreased ROS levels compared with the LV-down group.

### 3.9. SIRT1 Knockdown Enhanced Activation of the NLRP3 Inflammasome by Regulating ROS

**Figure 9:**
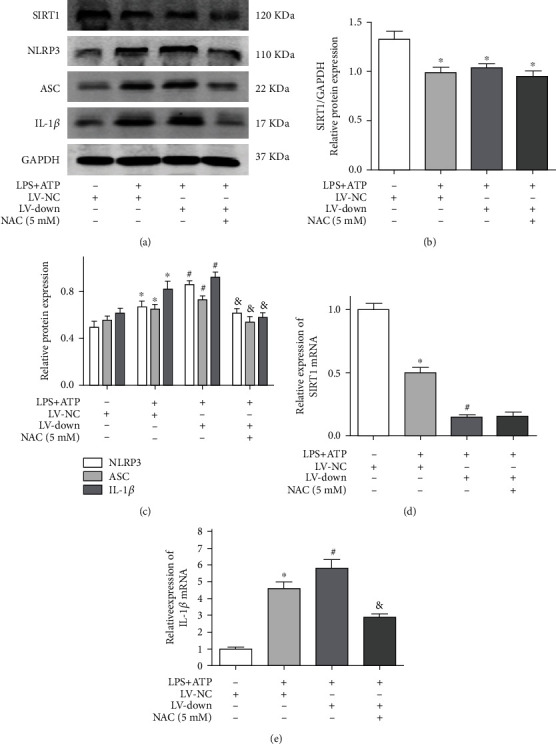
SIRT1 knockdown enhanced the activation of NLRP3 inflammasome by regulating ROS. Cells were divided into four groups: LV-NC, LV-NC+10 *μ*g/ml LPS+4 mM ATP, LV-down+10 *μ*g/ml LPS+4 mM ATP, and LV-down+10 *μ*g/ml LPS+4 mM ATP+NAC (5 mM); (a) the protein expression of SIRT1, NLRP3, ASC, and IL-1*β* was detected by western blotting; (b) the quantitative blots of SIRT1; (c) the quantitative blots of NLRP3, ASC, and IL-1*β*; (d) the gene expression of SIRT1; (e) the gene expression of IL-1*β*. ^∗^*P* < 0.05 versus LV-NC. ^#^*P* < 0.05 versus LV-NC+10 *μ*g/ml LPS+4 mM ATP. ^&^*P* < 0.05 versus LV-down+10 *μ*g/ml LPS+4 mM ATP. *n* = 5 per group. The data represent the means ± SD.

To further assess the role of ROS in the activation of the NLRP3 inflammasome in SIRT1 knockdown conditions, we analyzed the protein expression of SIRT1, NLRP3, ASC, and IL-1*β*. The results showed that the LV-down group further increased NLRP3 inflammasome proteins compared with the LV-NC group by LPS and ATP treatment. In addition, the expression of SIRT1 in the LV-down plus LPS+ATP group and LV-NC plus LPS+ATP group was decreased compared with that in the LV-NC group (Figures 9(a) and 9(b)). However, there was no difference between the LV-down plus LPS+ATP group and the LV-NC plus LPS+ATP group. Furthermore, the LV-down plus NAC group showed an obvious reduction in the NLRP3 inflammasome proteins compared with the LV-down group (Figures 9(a) and 9(c)). The mRNA levels of SIRT1 and IL-1*β* were measured in each group. The results showed that the LV-down group could further decrease SIRT1 expression compared with the LV-NC group (Figure 9(d)). In addition, the LV-down plus NAC group showed an obviously reduced IL-1*β* expression compared with the LV-down group (Figure 9(e)).

### 3.10. High Levels of Ammonia Increased ROS Levels and Induced Apoptosis in HT22 Neuronal Cells

**Figure 10:**
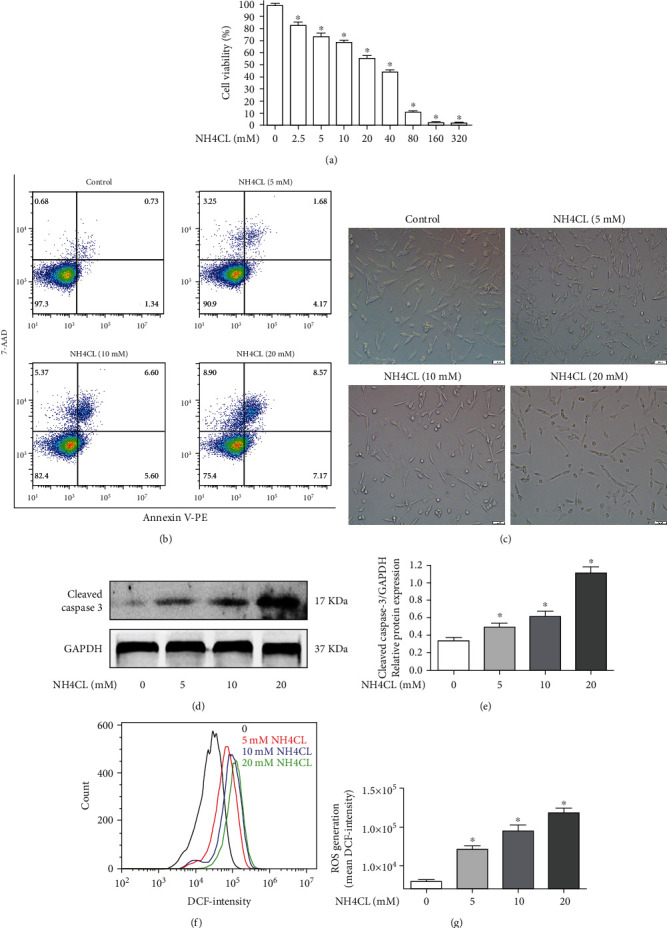
High levels of ammonia increased the ROS levels and induced the apoptosis in HT22 neuronal cells. (a) The cell viability of HT22 cells at different concentrations NH_4_Cl (2.5, 5, 10, 20, 40, 80, 160, and 320 mM) was detected by CCK-8 assay; (b) the apoptosis rates were determined by flow cytometric analysis; (c) the morphological changes of HT22 cells; (d) cleaved caspase-3 was analyzed by western blotting; (e) the quantitative blots of cleaved caspase-3; (f) cells were treated with NH_4_Cl (0, 5, 10, and 20 mM); then, the ROS levels were measured by DCF fluorescence with flow cytometry; (g) the mean DCF-intensity in each group. ^∗^*P* < 0.05, compared with the control group. *n* = 5 per group. The data represent the means ± SD.

We explored the cytotoxicity of NH4CL on the viability of HT22 neuronal cells at different concentrations (2.5, 5, 10, 20, 40, 80, 160, and 320 mM) by CCK-8 assay. The results showed that NH_4_Cl suppressed the viability of HT22 cells in a dose-dependent manner (Figure 10(a)). Next, the apoptosis rates of HT22 cells treated with different dosages of NH_4_CL (0, 5, 10, and 20 mM) were determined by flow cytometric analysis. We found that NH_4_Cl induced the apoptosis of HT22 cells in a dose-dependent manner (Figure 10(b)). Morphological changes in HT22 cells were observed by light microscope in each group (Figure 10(c)). The apoptosis protein cleaved caspase-3 was analyzed by western blotting. The expression of cleaved caspase-3 was significantly increased by 10 and 20 mM NH_4_Cl (Figures 10(d) and 10(e)). Finally, we measured the intracellular ROS levels by using DCFH-DA in HT22 cells treated with different concentrations of NH_4_Cl. The results showed that high levels of ammonia significantly increased the ROS levels in HT22 cells (Figures 10(f) and 10(g)).

### 3.11. CAY10602 Reduced the Neurotoxicity of Ammonia in HT22 Cells

**Figure 11:**
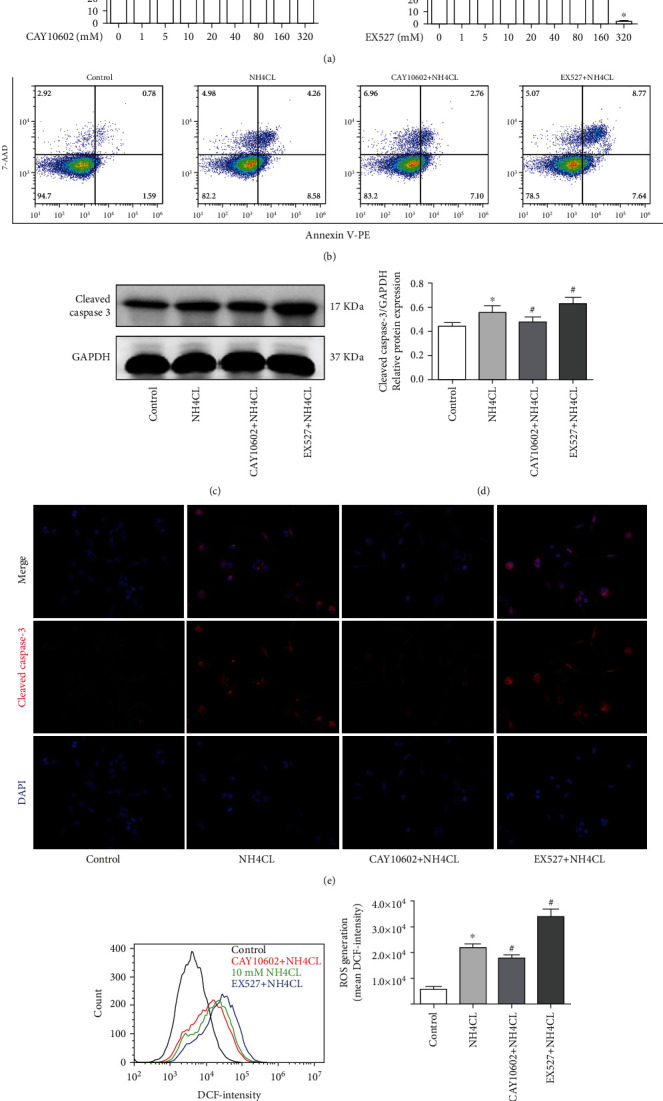
CAY10602 reduced the neurotoxicity of ammonia on HT22 cells. (a) The cytotoxicity of CAY10602 or EX527 on the cell viability of HT22 cells was detected by CCK-8 assay; (b) the apoptosis rates were determined by flow cytometric analysis in each group; (c) cleaved caspase-3 was analyzed by western blotting; (d) the quantitative blots of cleaved caspase-3; (e) immunofluorescence staining of cleaved caspase-3; (f) after treatment, the ROS levels were measured by DCF fluorescence with flow cytometry; (g) the mean DCF-intensity in each group. ^∗^*P* < 0.05, compared with the control group. ^#^*P* < 0.05, compared with the NH4CL group. *n* = 5 per group. The data represent the means ± SD.

First, we explored the cytotoxicity of CAY10602 or EX527 on the viability of HT22 cells at different concentrations by CCK-8 assay. According to the CCK-8 results, CAY10602 (10 *μ*M) and EX527 (20 *μ*M) were used for HT22 cell experiments (Figure 11(a)). Next, the results of flow cytometric analysis showed that CAY10602 administration reduced the apoptosis rates of HT22 cells in 10 mM NH_4_Cl, while EX527 increased cell apoptosis rates after exposure to NH_4_Cl (Figure 11(b)). In addition, the protein expression of cleaved caspase-3 was measured by western blotting and immunofluorescence staining. The results showed that CAY10602 reduced the expression of cleaved caspase-3, and that EX527 increased its protein expression (Figures 11(c)–11(e)). Finally, we measured the intracellular ROS levels in each group. The results showed that CAY10602 decreased the ROS levels in HT22 cells, and that EX527 increased the ROS levels (Figures 11(f) and 11(g)).

## 4. Discussion

**Figure 12:**
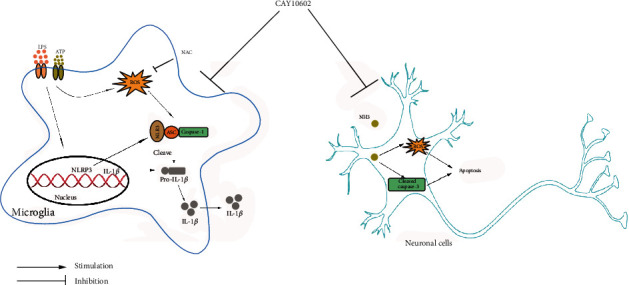
A schematic showing the protective effect of CAY10602 on microglia and neuronal cells. The activation of NLRP3 inflammasome complex involves two signals: LPS and ATP. LPS and ATP stimulation results in the formation of NLRP3 inflammasomes and ROS accumulation. CAY10602 alleviates the activation of NLRP3 inflammasome and ROS in BV2 microglia cells. CAY10602 alleviates the neurotoxicity of ammonia on HT22 neuronal cells.

In the present study, we explored the underlying mechanisms and effects of SIRT1 on thioacetamide-induced HE rats. We found that CAY10602 alleviated liver injury and neuroinflammation in HE rats. Furthermore, CAY10602 inhibited NLRP3 inflammasome activation in BV2 microglial cells. Additionally, the main finding was that CAY10602 reduced the neurotoxicity of NH_4_Cl in HT22 neuronal cells (Figure 12).

As described previously, several factors are involved in the pathogenesis of HE, including neuroinflammation, oxidative stress, and hyperammonemia. Recent studies have indicated the important role of neuroinflammation in the pathogenesis of HE [23]. In the brain, activated microglia are the main source of proinflammatory cytokines, which contribute to the development of neuroinflammation [24]. A previous study found that expression of the cortical microglial marker Iba-1 was significantly higher in HE rats than in the control group [25]. Similarly, other studies reported that activation of microglia was observed in HE models [26, 27]. Our study also showed the activation of microglia in the cortex and hippocampus. Furthermore, oxidative stress plays an important role in the pathogenesis of HE. The level of brain oxidative stress marker MDA was significantly higher in the HE groups after TAA administration [28]. Several studies have demonstrated oxidative stress injury in the brain tissues of HE rat models [29, 30]. Consistent with these findings, our study also confirmed elevated brain oxidative stress markers in the brain tissues of HE rats. In addition, we found that CAY10602 alleviated oxidative stress injury in the brains of HE rats. Similar to this finding, a recent study showed that the SIRT1 inhibitor EX527 increases ROS production in renal tissues in septic mice [31].

In the present study, the HE rat model presented obvious liver histological injury, high levels of serum ammonia, neurological decline, and microglial activation. In addition, our results indicated that CAY10602 significantly alleviated these injuries in HE rats while EX527 increased ammonia and microglial activation. The results were consistent with a previous study, which showed the protective effect of resveratrol on neurological deficits in HE rats [10]. To explore the exact regulatory mechanisms, the relative signaling pathways and cell types were measured. Our data showed that CAY10602 inhibited NLRP3 inflammasome activation and mainly decreased the expression of NLRP3 in microglia in HE brain tissue.

Based on the above findings, it is suggested that microglial activation plays an important role in the development of HE. In addition to the effect of CAY10602 on liver injury, neurological effects were also detected. We assessed the effect of SIRT1 on NLRP3 inflammasome activation in vitro. BV2 cells have been extensively used in studies related to neuroinflammation and neurodegeneration. Many studies have demonstrated that BV2 cells could be a valid substitute for primary microglia in cell experiments [32]. An increasing number of researches have demonstrated that SIRT1 is closely linked with the activation of the NLRP3 inflammasome [9, 33]. To further confirm the effect of SIRT1 on NLRP3 inflammasome activation in vitro, a model of NLRP3 inflammasome activation in BV2 microglial cells was established by using LPS and ATP. CAY10602 administration inhibited the activation of the NLRP3 inflammasome in BV2 microglial cells. Moreover, EX527 or SIRT1 knockdown also enhanced the activation of the NLRP3 inflammasome. These results were consistent with one previous study, which showed that SIRT1-specific siRNA increases the activation of the NLRP3 inflammasome in vascular endothelial cells after LPS and ATP challenge [33]. In our previous article, we found the anti-inflammatory role of SIRT2 inhibitor AGK2 in LPS-induced BV2 cells and LPS-treated mice, and its mechanism might through suppressing MAPK signaling pathways [34]. It suggests that sirtuin family members play important role in inflammation aspect. These data showed that SIRT1 could downregulate the activation of the NLRP3 inflammasome.

There are three possible mechanisms of inflammasome activation: elevated ROS levels, K+ efflux, and lysosomal rupture [35]. ROS generation has been shown to be a critical mechanism triggering NLRP3 inflammasome activation in response to DAMPs [36]. In support of the activation mechanism, the ROS inhibitor NAC decreased the activation of the NLRP3 inflammasome in alveolar macrophages exposed to serum from burn-injured animals [37]. Another study reported that NAC inhibits the expression of NLRP3, IL-1*β*, and IL-18 in HK2 cells after albumin stimulation [38]. In our study, ROS levels were increased with the degree of activation of the NLRP3 inflammasome. In addition, NAC pretreatment significantly reduced the high levels of ROS induced by LPS and ATP treatment. Finally, we further explored the relationship among SIRT1, ROS, and the NLRP3 inflammasome. The results showed that the SIRT1-LV-down group exhibited increased activation of the NLRP3 inflammasome compared with the LV-NC group, while compared with the SIRT1-LV-down group, pretreatment with NAC alleviated the degree of activation of the NLRP3 inflammasome. Taken together, our data indicated that downregulation of SIRT1 mediated the activation of the NLRP3 inflammasome by elevating ROS in BV2 microglial cells. Consistent with this study, resveratrol reduced ROS production and NLRP3 activation in a SIRT1-dependent traumatic brain injury rat model [39]. Based on these data, it is suggested that SIRT1 could inhibit the activation of the NLRP3 inflammasome by downregulating ROS.

In addition to neuroinflammation, hyperammonemia is another mechanism in the development of HE. High levels of ammonia are toxic to the brain, which contributes to the abnormal alteration of neurotransmitters and glutamate. The direct toxicity of excess ammonia on neurons has been reported [4]. In our study, to assess the protective effect of CAY10602 on the neurotoxicity of ammonia in neuronal cells, NH_4_Cl-induced HT22 neuronal cells were used to simulate a hyperammonemia cell model. Our results showed that CAY10602 alleviated the neurotoxicity of ammonia in HT22 cells by inhibiting ROS and cell apoptosis. Consistent with this research, ammonia induced ROS production and cell apoptosis in neurons [5].

In conclusion, our study suggested that CAY10602 alleviated microglial activation in the brain by suppressing the NLRP3 inflammasome. In addition, CAY10602 reduced the neurotoxicity of ammonia in HT22 cells. Overall, this study provides a better understanding of the mechanisms by which SIRT1 regulates NLRP3 inflammasome activation in HE. However, the specific target by which SIRT1 regulates the NLRP3 inflammasome needs further research. We will further confirm the effect of SIRT1 in HE by using Sirt6-KO mice and transgenic mice in the future.

## Data Availability

The data used to support the findings of this study are available from the corresponding author upon request.

## Abbreviations

7-AAD: 7-Amino-actinomycin D
ALT: Alanine aminotransferase
ANOVA: Analysis of variance
ASC: Apoptosis-associated speck-like protein containing a CARD
AST: Aspartate aminotransferase
ATP: Adenosine triphosphate
BCA: Bicinchoninic acid
BSA: Bovine serum albumin
DAMP: Damage-associated molecular pattern
DAPI: 4′,6-Diamidino-2-phenylindole
DMSO: Dimethylsulphoxide
EGFP: Enhanced green fluorescent protein
ELISA: Enzyme-linked immunosorbent assay
GAPDH: Glyceraldehyde-3-phosphate dehydrogenase
GSH: Glutathione
H2O2: Hydrogen peroxide solution
Iba-1: Ionized calcium-binding adaptor molecule 1
IL-1*β*: Interleukin-1*β*
IL-6: Interleukin-6
iNOS: Inducible nitric oxide synthase
LPS: Lipopolysaccharide
LV: Lentiviral vector
MDA: Malondialdehyde
NADPH: Nicotinamide adenine dinucleotide phosphate
NH4Cl: Ammonium chloride
NLRP3: NOD-like receptor containing a pyrin domain 3
PAMP: Pathogen-associated molecular pattern
PBS: Phosphate buffered saline
PRR: Pattern recognition receptors
PVDF: Polyvinylidene fluoride
ROS: Reactive oxygen species
TAA: Thioacetamide
TLR: Toll-like receptor
TNF-*α*: Tumor necrosis factor-*α*.

## Supplementary Materials

Supplementary Materials Supplementary 1: Figure S1: effect of CAY10602 on liver histological changes in HE female rats.
