# Supplementary material for: Role of SIRT1 in Hepatic Encephalopathy: In Vivo and In Vitro Studies Focusing on the NLRP3 Inflammasome
Source: Oxid Med Cell Longev. 2021 Oct 12;2021:5522708. doi: 10.1155/2021/5522708 (PMC8526203; doi:10.1155/2021/5522708)
Supplement: Supplementary Materials — Supplementary 1: Figure S1: effect of CAY10602 on liver histological changes in HE female rats. [file 5522708.f1.doc]

Supplementary materials

A


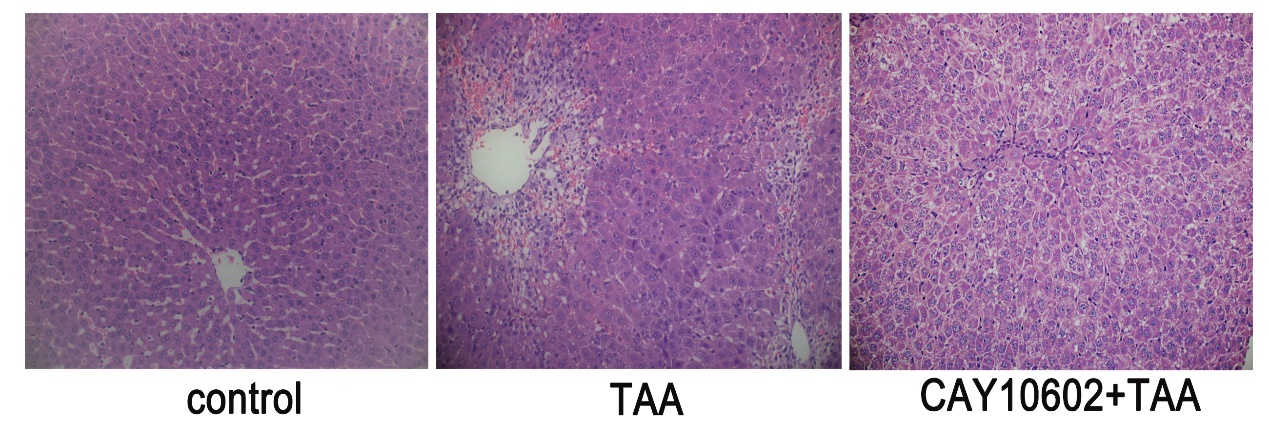


B


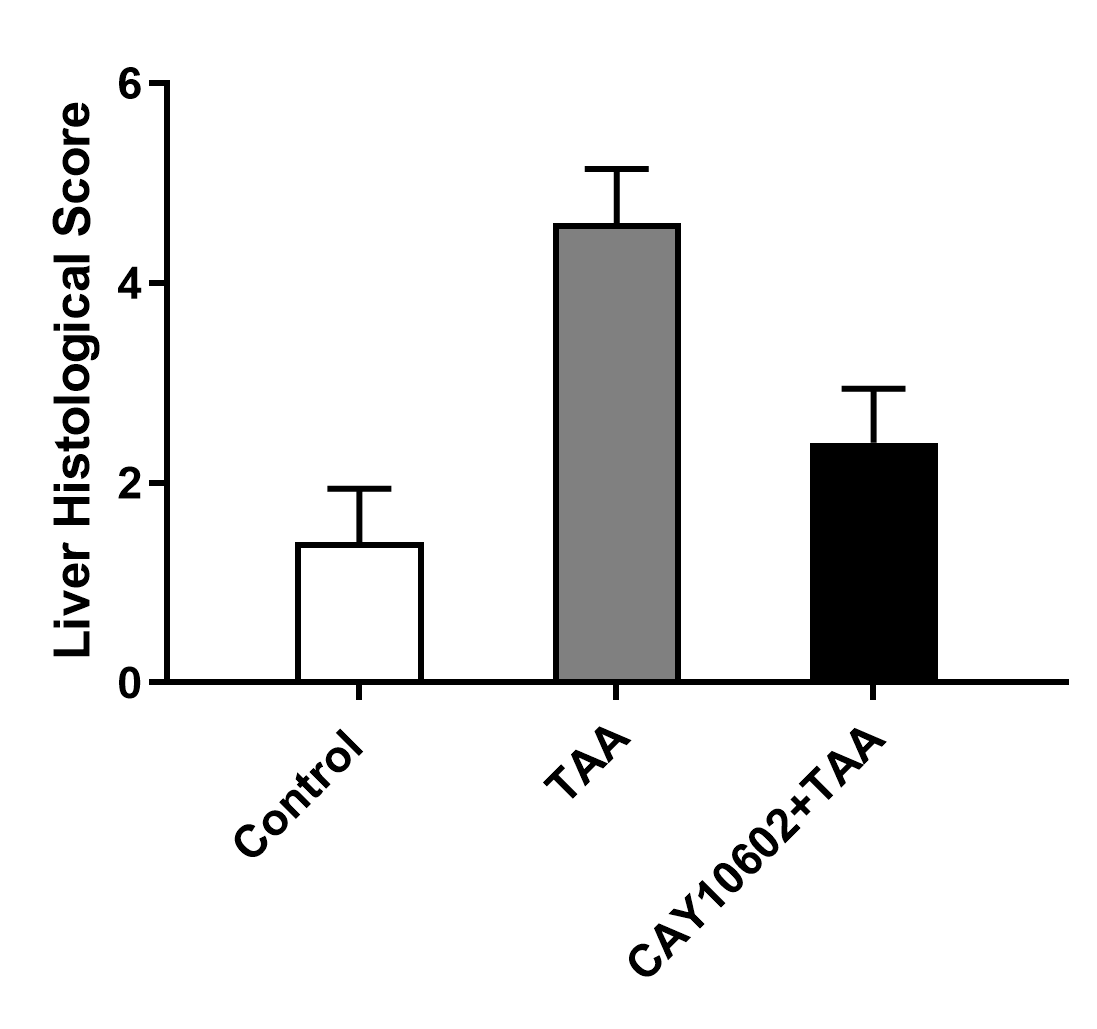


Figure S1. Effect of CAY10602 on liver histological changes in HE female rats. (a)The histological changes of liver were observed by HE staining. (b) The liver histological score of liver. n=5 per group. The data represent the means ± SD. P < 0.05, compared with the control group. #P < 0.05, compared with the TAA group.

Supplementary methods

Female Wistar rats (5-6 weeks, 130-180g) were obtained from Beijing vital river laboratory animal technology. All animal use and care protocols were approved by the committee on the ethics of animal experiments of Remmin Hospital of Wuhan University (certificate numbers: SYXK (E) 2015– 0027) and performed in compliance with national institutes of health (NIH) guide for the care and use of laboratory animals. In this study, HE induced by TAA was extensively proved to be a well-characterized animal model of HE. Briefly, the female rats were randomly divided into three groups (n=5 each): control, TAA, CAY10602 plus TAA group.
